# Mercury dynamics in surface-active soil invertebrates from an ecological reserve in the Brazilian savanna

**DOI:** 10.1007/s10661-026-16051-0

**Published:** 2026-10-20

**Authors:** Iara Oliveira Fernandes, Lucas Cabrera Monteiro, Vinícius Lima de Miranda, João Pedro Rudrigues de Souza, Ygor Oliveira Sarmento Rodrigues, Ésio de Castro Paes, William Fernando Antonialli Júnior, José Vicente Elias Bernardi

**Affiliations:** 1 Graduate Program in Environmental Sciences, Faculty UnB Planaltina, University of Brasília, Brasília, Distrito Federal 73345-010 Brazil; 2 Graduate Program in Ecology, Institute of Biological Sciences, University of Brasília, Brasília, Distrito Federal 70910-900 Brazil; 3 Graduate Program in Zoology, Institute of Biological Sciences, University of Brasília, Brasília, Distrito Federal 70910-900 Brazil; 4 Graduate Program in Chemistry, Institute of Chemistry University of Brasília, Brasília, Distrito Federal 70910-900 Brazil; 5 Graduate Program in Soils and Plant Nutrition, Federal University of Viçosa, Viçosa, Minas Gerais 36570-900 Brazil; 6 Behavioral Ecology Laboratory (LABECO), Natural Resources Study Center (CERNA), Mato Grosso Do Sul State University, Dourados, Mato Grosso do Sul 79804-970 Brazil

**Keywords:** Bioaccumulation, Litter, Feeding habit, Biomagnification, Organic matter

## Abstract

This study evaluated total mercury (THg) distribution and accumulation in soil, litter, and surface-active soil invertebrates at the Águas Emendadas Ecological Station (ESECAE), a savanna reserve in central Brazil. We assessed whether THg differed between soil and litter; whether soil pH and organic matter influenced soil THg, the degree of THg accumulation in invertebrates relative to soil and litter; whether feeding habits structured epigeic soil fauna according to THg concentrations; and whether biomagnification could be inferred within the local trophic network. THg concentrations were significantly higher in litter (51.7 ± 5.6 ng g⁻^1^) than in soil (34.9 ± 13.5 ng g⁻^1^; *t* = 4.402, df = 7, *p* = 0.0032). Soil organic matter was positively associated with soil THg (*R*^2^ = 0.455, *p* = 0.039), whereas pH showed no significant relationship. Invertebrate THg concentrations ranged from 0.095 to 970.70 ng g⁻^1^, reaching values approximately 15 times higher than those in soil or litter. Overall, 83.75% of samples showed bioaccumulation potential, and macroaccumulation predominated relative to both soil (80.38%) and litter (68.53%). Grouping by feeding habits revealed consistent accumulation patterns, indicating trophic-level biomagnification. Predator frequency increased from 9.1% in the lowest THg group to 45.5% in the highest, while phytophagous organisms decreased from 26.1% to 2.3%. Our results underscore the complexity of mercury cycling in terrestrial food webs and highlight the role of ecological traits and environmental parameters in modulating mercury bioavailability and transfer.

**Supplementary Information:**

The online version contains supplementary material available at https://doi.org/10.1007/s10661-026-16051-0.

## Introduction

The increasing exploitation of natural resources has intensified the release and accumulation of chemical pollutants in ecosystems (Bayabil et al., 2022). Among the pollutants associated with this growing environmental pressure, mercury (Hg) is of particular concern because of its toxicity, persistence, and global mobility. Although Hg occurs naturally, anthropogenic activities, especially artisanal gold and coal mining and fossil fuel combustion, have significantly elevated its environmental burden (Xiao et al., 2022; Wang et al., 2021). In the environment, Hg can transform into methylmercury (MeHg), a highly mobile and bioaccumulative neurotoxin, leading to adverse ecological and human health effects (Sahakyan et al., 2019; Charbonneau et al., 2022). To address this threat, the Minamata Convention was established as a global treaty to reduce Hg emissions, production, and use (Fernandes et al., 2021; Gustin et al., 2016).

Mercury moves through the environment by a complex biogeochemical cycle involving multiple environmental compartments. It can be found in the atmosphere, hydrosphere, and pedosphere, and can accumulate specifically in sediments, soil, and soil biota (Al-Sulaiti et al., 2022; Wang et al., 2021). In soil, Hg dynamics may pose ecological and human health risks, as the accumulation of mercury in this compartment affects the edaphic community, given its bioaccumulation in terrestrial insect tissues (Borozan et al., 2021; Huang et al., 2021; Zhang et al., 2012). As a consequence, the diversity of these organisms can be reduced, especially in contaminated areas, due to the high concentration of Hg in the environment (Anshary et al., 2018). Furthermore, soil organic matter (SOM) plays a dual role in Hg dynamics: it can strongly bind inorganic Hg via reduced sulfur functional groups, whereas, under favorable redox and microbial conditions, it can also promote Hg mobilization and methylation into methylmercury (MeHg), facilitating its accumulation and transfer through terrestrial trophic levels (He et al., 2019).

Among the environmental compartments through which Hg circulates, vegetation plays an important role, as it regulates the fluxes of Hg° and Hg^2^⁺ by absorbing Hg and depositing it into soils via litterfall (Manceau et al., 2018; Zhou et al., 2018). This organic compartment has a close relationship with surface-active soil invertebrates, which play a key role in litter decomposition, nutrient cycling, water dynamics, and soil structure formation (Zagatto et al., 2019). Thus, mercury becomes part of the life cycle of these organisms, with traits such as feeding habits being key factors in mercury accumulation and flux in terrestrial food webs (Diouf et al., 2019; Yung et al., 2019). The accumulation of Hg in organism tissues can be assessed based on the food they consume or, in the case of soil organisms, on the environment in which they live (Cortet et al., 1999; Roy et al., 2021). Research in this area highlights the complexity of mercury accumulation in soil invertebrates and the various factors that can influence this process, including environmental conditions and biological interactions (Saxton et al., 2013; Tsui et al., 2019). Additionally, while the concentration and transfer of Hg through aquatic trophic chains have already been and continues to be widely studied (Dórea et al., 2023; Kolipinski et al., 2020; Moraes et al., 2023), research on Hg accumulation in soil organisms within terrestrial microhabitats remains incipient. This knowledge gap becomes even more evident in natural environments with no direct contamination sources, where data could contribute to understanding the aforementioned interactions (Fernandes et al., 2021).

In this context, the scarcity of studies on mercury dynamics in ecologically diverse environments such as savannas has driven researchers to collect data on mercury concentrations across different environmental compartments (Monteiro et al., 2021, 2024; Moraes et al., 2023). Recent studies have indicated the impact of land use on mercury concentrations in ecological reserves, especially those located within major urban centers (Dórea et al., 2023; Rodrigues et al., 2022). The Águas Emendadas Ecological Station (ESECAE), located in the Brazilian Midwest, is one of the most important natural reserves in the Federal District, where the unique phenomenon of the convergence of two major Latin American hydrographic basins, Platina and Tocantins/Araguaia, takes place.

This conservation unit is vulnerable to surrounding anthropogenic influences because it is bordered by urban and agricultural landscapes. Recent studies conducted at ESECAE have shown that soils and vegetation adjacent to roads contain higher Hg concentrations than those in the interior of the ecological station, a pattern attributed to vehicle emissions from paved roads (Pedrosa et al., 2026; Rodrigues et al., 2022). As there is no history of mining or industrial activities involving Hg in the surrounding area, vehicle emissions and regional atmospheric deposition are considered the primary sources of Hg in the region. The direct movement of Hg through road surface runoff is potentially more harmful to the soil than atmospheric deposition, as the hydrolysis process further facilitates Hg^2^⁺ adsorption by colloidal minerals (Zuo et al., 2013). Thus, understanding the source-receptor relationships associated with Hg emissions, transformations, and cycles in each environmental compartment is crucial to minimizing the risks of mercury pollution and exposure (Wang et al., 2021). Additionally, wildfires represent an important source of atmospheric Hg emissions in fire-prone biomes (Fisher et al., 2023). Supporting this, a recent study integrating laboratory heating experiments with regional emission estimates demonstrated that wildfires in ESECAE released approximately 2.6–3.2 kg of Hg over the past decade (Monteiro et al., 2026). However, despite these recent advances, Hg accumulation in terrestrial organisms within ESECAE has not yet been investigated.

The present study aims to investigate the distribution of mercury in the environmental compartments, soil, litter, and surface-active soil invertebrates at ESECAE, addressing the following questions: (i) Are there significant differences in THg levels between soil and litter compartments? (ii) Do soil chemical characteristics such as pH and organic matter (OM) influence THg concentrations in soil? (iii) What is the degree of THg accumulation in surface-active soil invertebrates relative to soil and litter? (iv) Can these invertebrates be grouped according to their THg concentrations and feeding habits? (v) Can we infer biomagnification of mercury in the trophic network of the epigeic soil fauna in the study area? We hypothesize that there are significant differences between THg levels in soils and litter and that the studied soil chemical characteristics influence THg concentration. We believe that groups sharing the same feeding habit and habitat can be grouped based on THg levels. Finally, we consider that feeding habits and THg concentrations establish a relationship that enables grouping these organisms and indicates that mercury undergoes biomagnification within terrestrial invertebrate trophic levels.

## Material and methods

### Study area

**Fig. 1:**
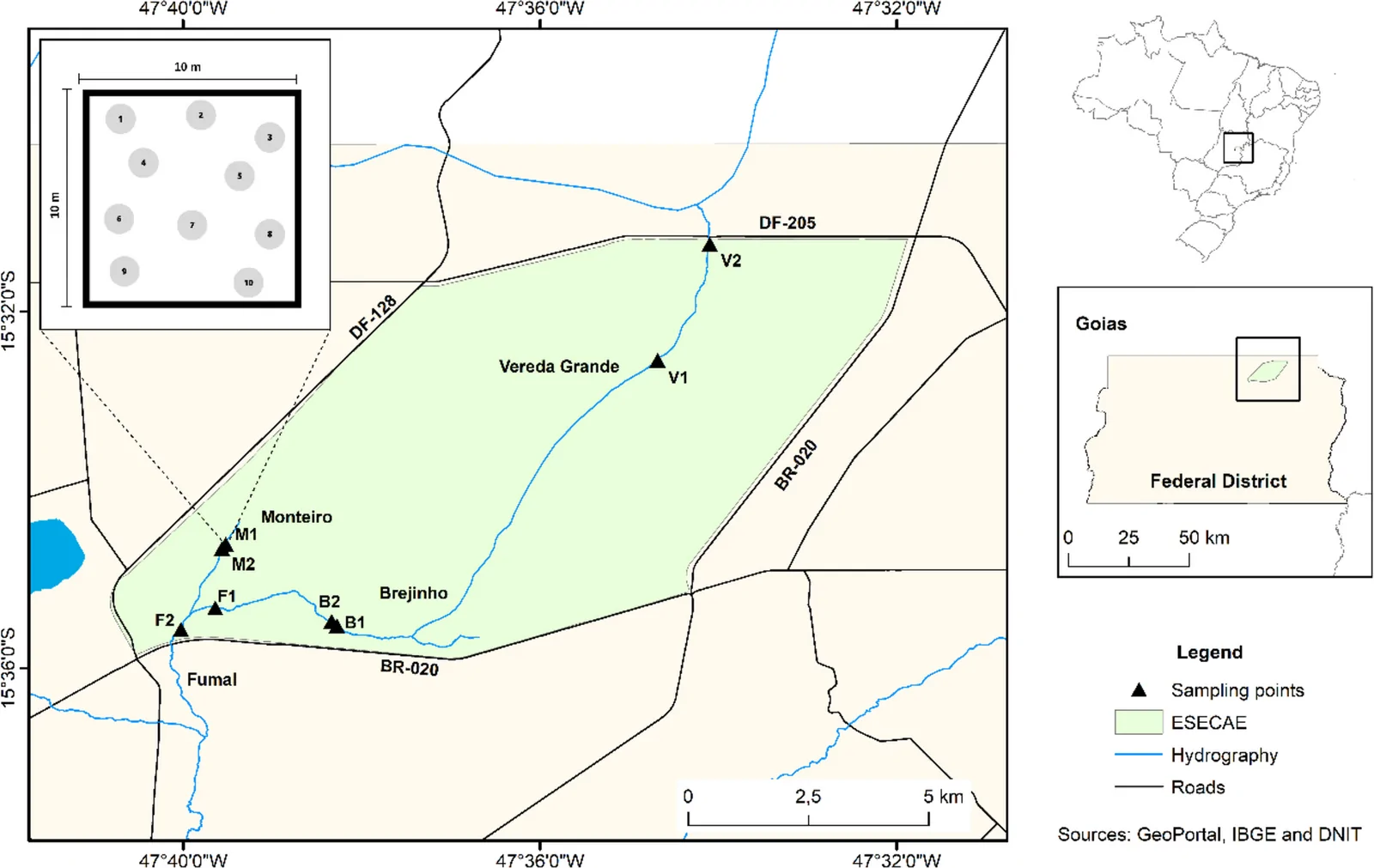
Map of the sampling points located in the Águas Emendadas Ecological Station (ESECAE), Federal District, Brazil

The Águas Emendadas Ecological Station (ESECAE) is an ecological reserve located in the Brazilian Cerrado, in the Administrative Region of Planaltina, Federal District (15° 31′ to 15° 36′ S and 47° 31′ to 47° 42′ W; elevation, 924–1177 m). An important peri-urban environmental reserve, ESECAE, supplies two major hydrographic basins in Latin America (Platina and Tocantins/Araguaia), encompassing an area of approximately 10,500 ha surrounded by urban and/or agricultural landscapes (Bagatini, 2006). The reserve’s annual precipitation ranges from 1200 to 1750 mm, with a climate characterized by well-defined seasonality between dry winters and rainy summers, classified as Cwa, Cwb, and Aw according to Köppen’s classification (Castro & Lima, 2020). The dominant soil types are Oxisols, with occurrences of Gleysols, Entisols, and Inceptisols (Lacerda, 2008). Figure 1 shows the extent of ESECAE, which contains the Vereda Grande stream draining northward into the Maranhão River basin, and the Brejinho, Monteiro, and Fumal streams that drain southward into the São Bartolomeu River basin (Lima and Silva, 2008). Sampling was limited to gallery forests, a distinct Cerrado physiognomy that follows watercourses across the Brazilian plateau (Ribeiro & Walter, 2021).

### Sample collection and processing

Surface-active soil invertebrates, soil, and litter samples were collected from eight sampling sites adjacent to the headwater streams Brejinho, Monteiro, Fumal, and Vereda Grande (Fig. 1). Each sampling site covered a 100 m^2^ area (10 × 10 m) and contained 10 pitfall traps (10 × 20 × 22 cm), whose combined catches constituted the composite sample for that unit. The traps were filled with 70% ethanol to euthanize and preserve the collected invertebrates. Traps remained in the field between May and July 2021 and were sampled weekly, resulting in seven sampling campaigns. The collected individuals were taxonomically identified using the entomology manual by Borror et al. (1989), counted, lyophilized, measured, and homogenized using an agate mortar and pestle (Text S1 and Fig. S1). Individuals were grouped at the lowest possible taxonomic level, ranging from order to subfamily. Litter and organic soil samples (0–20 cm) were collected at the center of each sampling unit and dried at 65 °C to constant weight. Litter samples were ground using an electric grinder, whereas soil samples were sieved to obtain the < 20 µm fraction.

### Determination of hg, organic matter, and ph

Total mercury (THg) concentration was determined by atomic absorption spectrometry using a RA-915+Lumex® instrument. The dried and ground solid samples were weighed on sampler quartz boats and directly injected into a pyrolysis chamber (Pyro-915+) for matrix decomposition and mercury atomization (Sholupov et al., 2004). THg quantification in soil and litter was performed by external calibration using different volumes of a diluted Hg^2+^ standard solution in 10% HNO_3_ (Fluka®), with a linear range of 0.21–80 ng. For arthropods, the analytical curve was prepared by weighing different masses of the PACS-3 estuarine sediment certified reference material, and the linear range was 0.06–19 ng. The detection limit was calculated from the standard deviation of seven blank measurements (a clean sampler boat with 100 uL of Nanopure® water, 18.0 ± 0.2 MΩ·cm^−1^) and ranged from 0.21 to 0.36 ng for soil and litter samples and from 0.06 to 0.34 ng for arthropod samples. Analytical accuracy was assessed using certified reference materials: NIST 1515 (Apple leaves), BCR 463 (Tuna fish), and NIST 2711a (Montana II soil). Additionally, we used an internal reference material, PXMD (Catfish muscle, Environmental Biogeochemistry Laboratory, Federal University of Rondônia). Recovery rates ranged from 81 to 103%.

Soil organic matter (SOM) content was determined based on organic carbon (C-org) content multiplied by a factor of 1.724 (Teixeira et al., 2017). C-org was measured via dichromate oxidation (0.167 mol·L^−1^ K₂Cr₂O₇) in an acidic medium (Walkley and Black method), using an external heat source to maximize oxidation (Yeomans & Bremner, 1988). Soil pH was measured in a soil:distilled water solution at a 1:2.5 ratio using a potentiometer with a combined glass electrode (Teixeira et al., 2017).

### Data analysis

Shapiro–Wilk (*n* < 50) or Kolmogorov–Smirnov (*n* > 50) tests were used to assess data normality. Comparison between THg concentrations in soil and litter was performed using a paired *t*-test (for normally distributed data). Linear regressions were used to evaluate the effect of organic matter and pH on THg concentrations in the soil. Assumptions of no autocorrelation (Durbin–Watson), normality (Shapiro–Wilk), and homoscedasticity (Breusch–Pagan) were met.

The bioaccumulation factor (BAF) was calculated as the ratio between THg concentrations in the organism (mg·kg^−1^, dry weight) and in the litter or soil (mg·kg^−1^, dry weight). BAF results were interpreted according to Roy et al. (2021), in which BAF < 1 indicates insignificant bioaccumulation; 1 ≤ BAF ≤ 2 indicates microaccumulation of Hg; and BAF > 2 indicates macroaccumulation. Next, we used classification tree algorithms and discriminant analyses to assess patterns of THg distribution among organisms with different feeding habits. A total of 443 samples were included in the statistical analyses. Taxa with only one occurrence, highly generalist feeding habits, or extreme THg values were excluded from the analyses to improve model stability and reduce the influence of sparse classes and outliers on the multivariate analyses. The classification tree was used to group feeding habits based on THg concentrations in organisms. Discriminant analysis was applied to separate organisms by feeding habit, using three quantitative descriptors: THg concentrations in organisms, mean organism size (Hm), and BAF calculated from THg concentrations in litter. A detailed description of the classification and discriminant analyses is available in the Supplementary Material (Text S2). A significance level of *p* < 0.05 was adopted for all analyses. Statistical procedures were carried out using XLSTAT Premium (Addinsoft, Paris, France), and the distribution of macro- and microaccumulator organisms was illustrated using simple graphs prepared in Prism 10.8 (GraphPad Software, San Diego, USA).

## Results

### Soil and litter

**Fig. 2:**
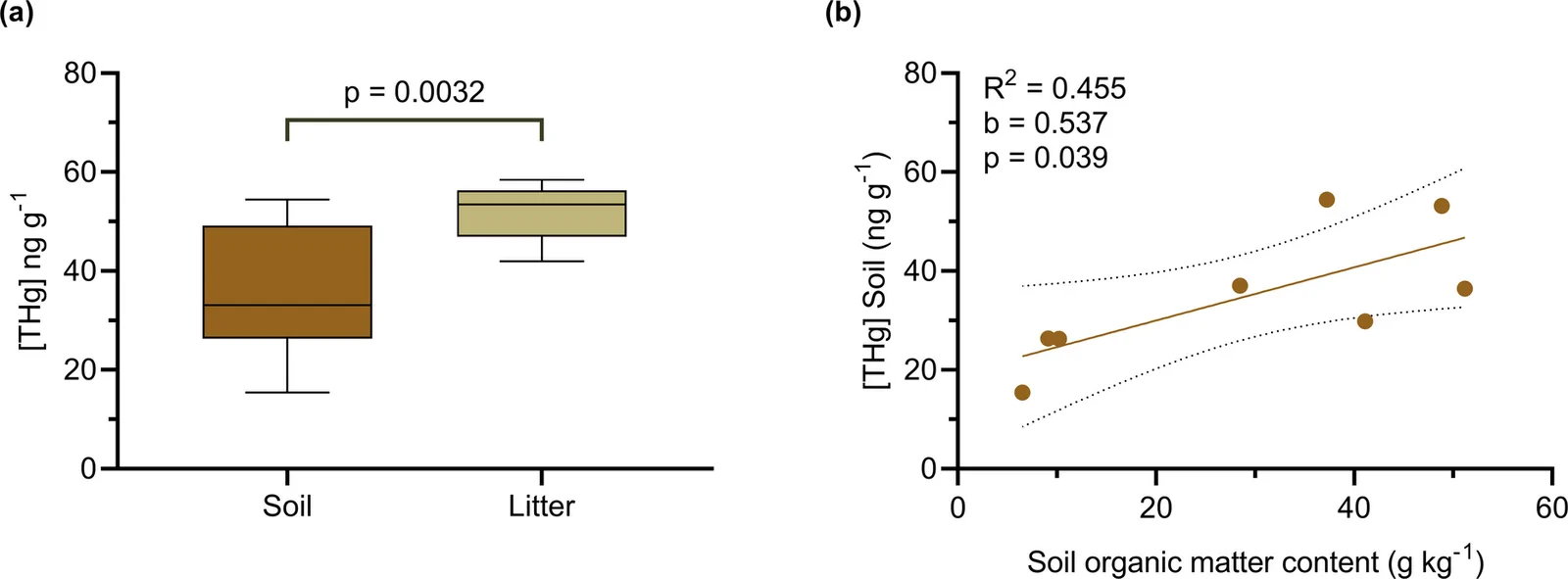
**a** Comparison of THg concentrations between soil and leaf litter and **b** linear regression between THg concentrations and soil organic matter content

THg concentrations were significantly higher in the litter (51.7 ± 5.6 ng g⁻^1^) compared to soil (34.9 ± 13.5 ng g⁻^1^) (*t* = 4.402, df = 7, *p* = 0.0032) (Fig. 2a). Linear regression showed that soil organic matter content significantly predicted soil THg concentrations (*R*^2^ = 0.455, *b* = 0.537, *p* = 0.039; Fig. 2b). The estimated slope indicates that THg concentrations increased by 0.537 units for each unit increase in soil organic matter content. No significant relationship was observed between THg and soil pH.

### Surface-active soil invertebrates

A diverse array of surface-active soil invertebrates was identified in the Águas Emendadas Ecological Station (ESECAE), totaling 15 orders. The order Hymenoptera was the most frequent in the collections, present in 136 of the 493 samples analyzed. In contrast, praying mantises (Orthoptera: Mantodea; 182.86 ng g⁻^1^), water scavenger beetles (Coleoptera: Hydrophilidae; 168.46 ng g⁻^1^), Lantern flies  (Hemiptera: Fulgoridae; 29.22 ng g⁻^1^), and ants belonging to the subfamilies Dolichoderinae (330.25 ng g⁻^1^) and Pseudomyrmicinae (50.42 ng g⁻^1^) were each represented by a single sample.

The highest average THg concentrations among orders were observed in termites (Isoptera, 458.11 ± 357.9 ng g⁻^1^), followed by scorpions (Scorpiones, 457.48 ± 264.06 ng g⁻^1^), and spiders (Araneae, 340.70 ± 184.72 ng g⁻^1^); the lowest was in cicadas and bedbugs (Hemiptera, 141.45 ± 138.58 ng g⁻^1^). Marked variability in THg concentrations was observed across taxa, with some groups exhibiting standard deviations greater than the mean. This pattern was observed in harvestmen (Opiliones, 165.48 ± 232.36 ng g⁻^1^), flies (Diptera: Brachycera, 344.70 ± 390.36 ng g⁻^1^), burrowing bugs (Hemiptera: Cydnidae, 84.89 ± 97.40 ng g⁻^1^), wasps of the families Vespidae (590.14 ± 692.45 ng g⁻^1^) and Pompilidae (161.70 ± 176.90 ng g⁻^1^), crickets (Orthoptera: Gryllidae, 144.78 ± 179.76 ng g⁻^1^), and ants (Formicidae: Formicinae, 120.06 ± 123.90 ng g⁻^1^) (Table 1).

**Table 1:** Identification and mean THg concentration of surface-active soil invertebrates found in the Águas Emendadas Ecological Station (ESECAE)

Class	Order	Suborder^a^/family/subfamily^b^		THg (ng g^−1^)	Common name	Feeding habitats^c^	Ecological role^c^
Arachnida	^(46)^ Araneae	-		340.70 ± 184.16	Spiders	Predators	Regulatory effect on soil community; organic matter fragmentation; soil quality indicator
	^(3)^ Scorpiones			457.48 ± 264.06	Scorpions		
	^(18)^ Opiliones			165.48 ± 232.36	Harvestmen (night spiders)	Omnivore	
Insecta	^(36)^ Blattodea	**-**		183.13 ± 146.92	Cockroachs	Omnivores	Organic matter fragmentation
	^(92)^ Coleoptera	^(23)^ Carabidae		88.35 ± 79.67	Beetles	Predators or phytophagous	
		^(3)^ Cerambycidae		18.89 ± 14.34		Phytophagous	
		^(10)^ Chrysomelidae		79.53 ± 75.00			
		^(3)^ Curculionidae		68.44 ± 61.86			
		^(3)^ Meloidae		45.97 ± 19.81			
		^(1)^ Hydrophilidae		168.46		Phytophagous, predators, or necrophagous	
		^(2)^ Nitidulidae		48.18 ± 28.94		Generalistss	
		^(19)^ Scarabaeidae		303.96 ± 115.61		Necrophagous	
		^(28)^ Staphylinidae		182.75 ± 136.78		Generalist predators	
	^(14)^ Dermaptera	-		212.86 ± 187.53	Earwigs	Saprophagous or predators	
	^(26)^ Diptera	^(26)^ Brachycera^a^		344.70 ± 390.36	Fly	Predators or generalists	Regulatory effect on phytophagous insect community
	^(57)^ Hemiptera	^(3)^ Coreidae		102.68 ± 65.65	Cicadas and bedbugs	Phytophagous	Organic matter fragmentation
		^(3)^ Cicadidae		76.13 ± 14.48			
		^(29)^ Cydnidae		84.89 ± 97.40			
		^(1)^ Fulgoridae		29.22			
		^(2)^ Gelastocoridae		410.14 ± 320.45		Predators	
		^(19)^ Reduviidae		243.27 ± 156.88			
	^(136)^Hymenoptera	^(129)^ Formicidae	^(2)^ Ectatomminae^b^	113.60 ± 7.08	Ants	Generalist predators	Soil structure and transport of organic matter from the surface to deeper layers in the soil
			^(1)^ Dolichoderinae^b^	330.25		Omnivores	
			^(37)^ Formicinae^b^	120.06 ± 123.99			
			^(46)^ Ponerinae *Odontomachus*^b^	91.32 ± 78.05		Generalist predators	
			^(42)^ Myrmicinae	84.82 ± 83.82		Fungívores	
			^(1)^ Pseudomyrmicinae^2^	50.42			
		^(3)^ Vespidae		590.14 ± 692.45	Wasps	Generalist predators	
		^(4)^ Pompilidae		161.70 ± 176.90		Spider parasitoids	
	^(7)^ Isoptera	^(7)^ Termitidae		458.11 ± 357.91	Termites	Xylophagous	Soil structure
	^(4)^ Lepidoptera	-		212.80 ± 343.75	Moths	Phytophagous (sap suckers)	Plant and organic matter fragmentation
	^(1)^ Mantodea	-		182.86	Praying mantises	Predators	
	^(39)^ Orthoptera	^(39)^ Gryllidae		144.78 ± 179.76	Crickets	Omnivores	
	^(2)^ Phasmatodea	-		480.14 ± 312.65	Stick insects	Phytophagous	
^(7)^ Myriapoda	-	-		300.36 ± 143.89	Centipedes	Detritivores	Organic matter fragmentation and regulatory effect on soil community
Malacostraca	^(4)^ Isopoda	-		177.17 ± 94.05	Woodlice	Saprophagous and coprophagous	Organic matter fragmentation and soil structure

^a^Suborder classification

^b^Subfamily classification

^c^As described by the authors in the Text S3

*The subscript numbers shown on the left represent the number of samples from the corresponding group of organisms. Adult Diptera and Lepidoptera insects were collected

**Fig. 3:**
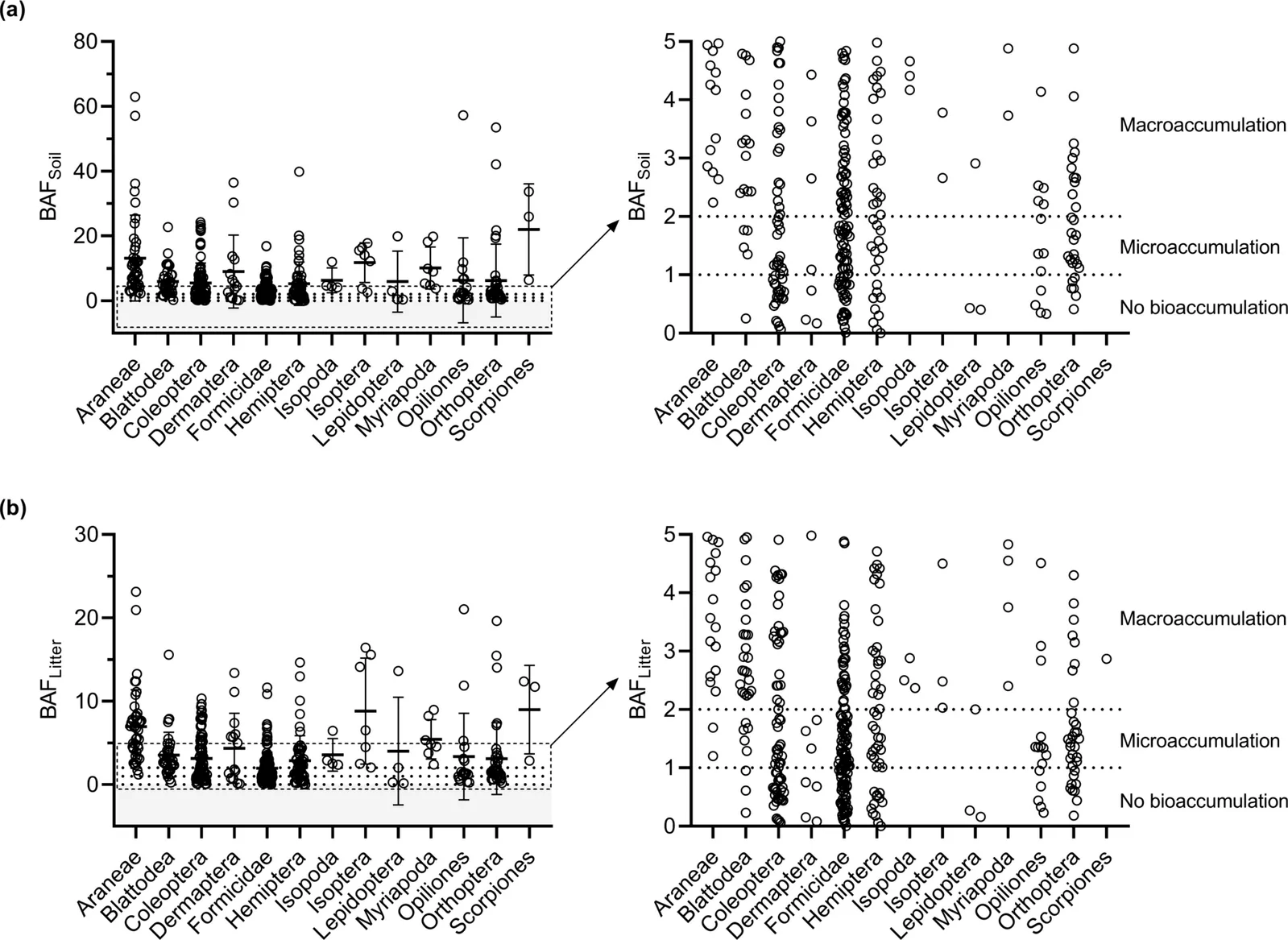
Bioaccumulation factor (BAF) based on THg concentrations in soil (**a**) and litter (**b**). The panels on the right show a zoomed-in view of BAF values between 0 and 5 to improve the visualization of bioaccumulation patterns, including no bioaccumulation (BAF < 1), microaccumulator (1 ≤ BAF ≤ 2), and macroaccumulator (BAF > 2)

The BAF based on soil (BAF_Soil_) ranged from 0.01 to 62.93 (mean 6.09 ± 8.22), while BAF based on litter (BAF_Litter_) ranged from 0.06 to 23.12 (mean 3.39 ± 3.60) (Fig. 3). Considering all the epigeic fauna samples analyzed, 83.75% showed bioaccumulation potential (BAF ≥ 1). Of this portion of samples with bioaccumulation potential, all showed bioaccumulation in relation to the soil (BAF_Soil_ ≥ 1) and 91.4% in relation to the litter (BAF_Litter_ ≥ 1). Regarding the organisms that showed bioaccumulation potential in relation to the soil, 80.38% were macroaccumulators (BAF_Soil_ ≥ 2), while in relation to the litter, 68.53% were macroaccumulators (BAF_Litter_ ≥ 2). The distribution pattern of the orders regarding BAF was similar across both environmental compartments (soil and litter). However, the family Formicidae was predominantly a macroaccumulator based on BAFSoil (71.57% of BAF_Soil_ ≥ 2), compared to BAF_Litter_ (50.59%). The spiders (Araneae) were predominantly macroaccumulators in both compartments (100% of BAF_Soil_ ≥ 2 and 95.45% of BAF_Litter_ ≥ 2).

**Fig. 4:**
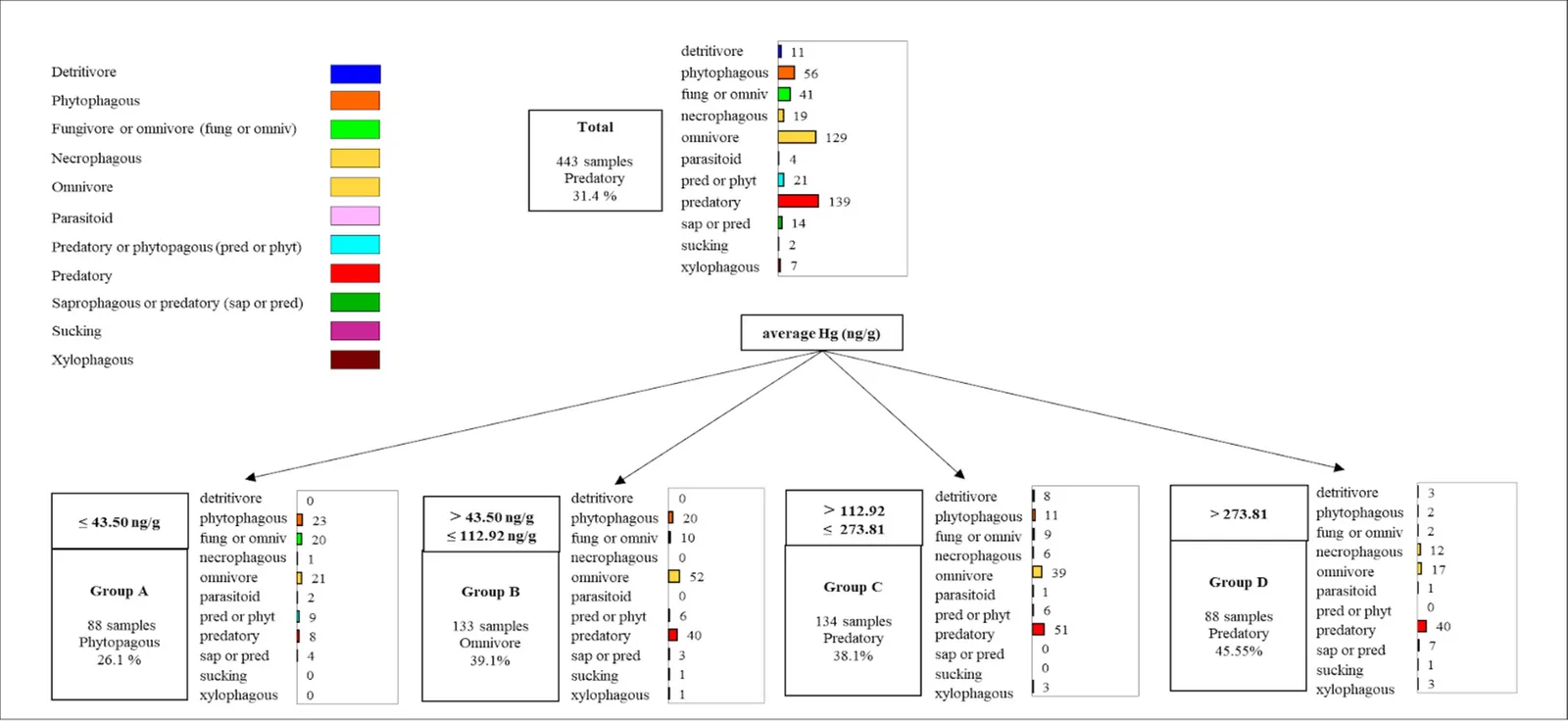
Grouping and frequency of surface-active soil invertebrates based on feeding habit and THg concentration in the Águas Emendadas Ecological Station, Brazil. The numbers indicated for each group/feeding habit correspond to the absolute number of samples analyzed. Pred or phyto, predatory or phytophagous; Sap or pred, Saprophagous or predatory

The grouping formed by the feeding habits of soil arthropods, within the THg amplitude of 970.60 ng g^−1^, resulted in four groups (Fig. 4): group A, ≤ 43.50 ng g^−1^; group B, > 43 50 ng g^−1^ and ≤ 112.92 ng g^−1^; group C, > 112.92 ng g^−1^ and ≤ 273.81 ng g^−1^; and group D, > 273.81 ng.g^−1^. Predators were the most frequent feeding group overall, representing 31.4% of the samples. Their relative frequency increased across the THg groups, from 9.1% in group A to 30.1% in group B, 38.1% in group C, and 45.5% in group D. In contrast, the frequency of phytophagous organisms decreased from 26.1% in group A to 15.0% in group B, 8.2% in group C, and 2.3% in group D. Omnivores predominated in group B (39.1%), whereas necrophagous organisms were more frequent in group D (13.6%) than in the other concentration groups.

**Fig. 5:**
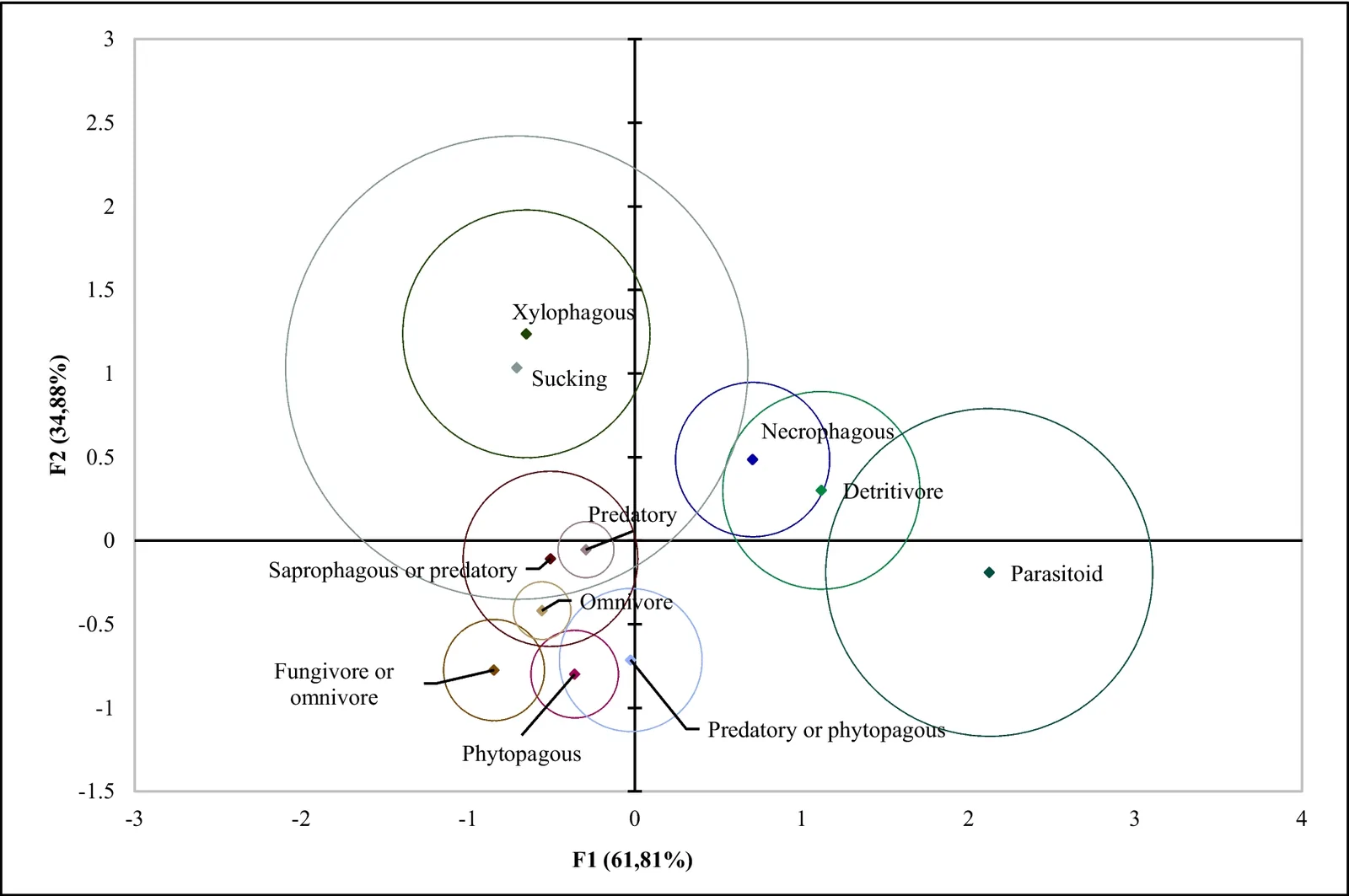
Feeding habit distribution of surface-active soil invertebrate groups from ESECAE in relation to mean body size, BAF_Litter_, and THg concentration

The multivariate profiles of the feeding habit groups, based jointly on THg concentration, BAF_Litter_, and mean body size (Hm), differed significantly (*p* < 0.0001; Fig. 5). The first two discriminant functions accounted for 96.69% of the total discrimination among feeding groups, with F1 explaining 61.81% and F2 explaining 34.88%. Xylophagous and sucking organisms occupied similar positions in the negative region of F1 and the positive region of F2 and did not differ significantly from each other (*p* > 0.05), although both were separated from most of the other feeding groups. Necrophagous and detritivorous organisms occupied the positive regions of F1 and F2 and showed partial overlap, whereas parasitoids exhibited the greatest separation along the positive F1 axis. Predatory and omnivorous organisms were positioned closer to the center of the discriminant space. Overall, xylophagous, sucking, necrophagous, detritivorous, and parasitoid organisms showed the greatest differentiation from the remaining feeding groups (*p* < 0.05).

## Discussion

### Mercury distribution in soil and litter

Our results indicated that THg concentrations were significantly higher in the litter compared to the soil; this is consistent with the role of forest canopies in intercepting atmospheric Hg and transferring it to the forest floor through litterfall. Atmospheric Hg° can be absorbed through leaf stomata, while oxidized and particulate forms can be retained on leaf surfaces and subsequently incorporated into litter (Ericksen et al., 2003; Rea et al., 2002; Wang et al., 2019). Opposite patterns were observed in studies conducted in the Atlantic Forest (Lima et al., 2017) and the Amazon (Richter et al., 2023), where higher concentrations were found in soil. However, a pattern similar to our study was found in a floodplain of the Cerrado biome affected by large-scale wildfires (Fernandes et al., 2024), as well as in a study conducted in forest ecosystems of the Atlantic Forest in Rio de Janeiro, which reported significantly higher concentrations in litter within a conservation unit impacted by industrial emissions (Buch et al., 2015). This comparison supports the atmospheric deposition–litterfall pathway as a plausible mechanism but does not imply that industrial emissions occur at ESECAE. In the study area, previous spatial investigations identified higher Hg concentrations in soils near highways and a decrease toward the reserve interior, indicating that road traffic represents a plausible diffuse anthropogenic source (Rodrigues et al., 2022). More recent analyses also identified elevated Hg concentrations in vegetation and soil near the boundaries of ESECAE and estimated an annual transfer of Hg from vegetation to soil through litterfall (Pedrosa et al., 2026). In this sense, fossil fuel combustion and dust deposition from highways may represent the main sources of potentially toxic elements, including Hg, for vegetation in areas with high traffic (Anwar et al., 2020; Pan et al., 2023).

Therefore, the difference in THg concentrations between litter and soil can be explained by potential Hg sources in the surrounding environment and characteristics of the sampling units. Natural contributions may include regional background atmospheric deposition and the weathering of parent materials, although previous mineralogical analyses at ESECAE indicated that the roadside Hg enrichment could not be explained exclusively by the mineral composition of the soil (Rodrigues et al., 2022). The surrounding land use pattern and the transport of Hg toward lower areas and watercourses have also been reported in previous studies conducted at ESECAE (Monteiro et al., 2021).

Our sampling units were in Gallery Forests, a typical Cerrado physiognomy composed of dense forest formations along watercourses. As such, the forest canopy acts as an interface between the atmosphere and terrestrial ecosystems, limiting direct atmospheric deposition onto the soil surface. Unlike other Cerrado physiognomies, Gallery Forests do not exhibit leaf abscission during the dry season (Ribeiro & Walter, 1998), resulting in prolonged exposure of leaves to atmospheric deposition. Additionally, the closed canopy reduces litter exposure to solar radiation and rainfall, thereby minimizing Hg losses through photoreduction and leaching processes (Richter et al., 2023). Therefore, prolonged atmospheric exposure combined with efficient retention and limited mobilization processes explain the higher Hg concentrations observed in litter compared to soil.

Linear regression results indicated a positive relationship between THg concentrations in the soil and organic matter content. This phenomenon is mainly due to Hg’s affinity for sulfur-containing functional groups in soil organic matter, such as thiol and disulfide, which promote sorption and oxidation of Hg° to Hg^2^⁺ (Gu et al., 2011). Plant debris and the biomass of fungi and bacteria are the main components of soil organic matter (Angst et al., 2021; Kögel-Knabner, 2002; Prescott & Vesterdal, 2021). In this regard, fungal necromass, which represents up to 40% of organic matter (Angst et al., 2021), may enhance Hg immobilization in soil (Maillard et al., 2023). Furthermore, Hg accumulation in litter can also lead to its transfer to the soil during decomposition (Zhou et al., 2018). Therefore, organic matter content is an important predictor of Hg concentrations in the soil of our study area.

### Surface-active soil invertebrates

THg concentrations in soil organism samples from ESECAE ranged from 0.095 to 970.70 ng g⁻^1^, a range 15 times greater than the THg levels found in soil (15.42 to 54.44 ng g⁻^1^; Fig. 2a) and litter (41.99 to 58.42 ng g⁻^1^; Fig. 2a). These results are consistent with studies conducted in savanna environments of the middle Araguaia River basin in Brazil, where average THg values in surface-active soil invertebrates were up to 20 times higher than the concentrations found in soil and litter (Fernandes et al., 2024). The bioaccumulation pattern was similar for soil and litter (Figs. 2 and 3), although THg-accumulating invertebrates via soil (100% of accumulators) were more representative than those accumulating via litter (91.4% of accumulators). Araneae was predominantly macroaccumulator in both compartments (Fig. 3), while Formicidae was mainly a macroaccumulator in soil (Fig. 3). These findings underscore the influence of edaphic organism–environment interactions on BAF, as previously noted by Yung et al. (2019), who stated that terrestrial invertebrate exposure to their environment can be a major source of Hg accumulation in some arthropods. For example, the orders Isoptera (Termitidae family, 458.11 ± 357.91 ng g⁻^1^), Scorpiones (457.48 ± 264.06 ng g⁻^1^), and Araneae (340.70 ± 184.16 ng g⁻^1^) exhibited the highest mean THg concentrations.

The high concentrations observed in spiders and scorpions, which are strictly predatory organisms, reflect Hg biomagnification through dietary intake, as demonstrated in recent studies in savanna ecosystems (Fernandes et al., 2024; Monteiro et al., 2024). In contrast, termites (Termitidae) and ants (Formicidae) exhibit strong interaction with the soil, where they live and construct their nests (Bruyn & Conacher, 1990; Diouf et al., 2019), increasing their exposure to Hg via environmental contact (Yung et al., 2019). Furthermore, the wide variation in Hg concentrations within Formicidae highlights the diversity of feeding strategies among subfamilies, ranging from fungivorous taxa (e.g., Myrmicinae and Pseudomyrmicinae) to generalist predators (e.g., Ectatomminae and Ponerinae). In this study, phytophagous organisms, particularly beetles (Coleoptera), were mainly composed of families such as Cerambycidae, Chrysomelidae, Curculionidae, and Meloidae (Marinoni et al., 2001; Paarmann et al., 2001), representing the base of the food web in the soil microhabitat. Thus, the life traits of soil arthropods are key factors in Hg accumulation and fluxes in terrestrial food webs. Herbivorous or phytophagous organisms that feed on living or dead leaves, fruits, flowers, or even wood (Brown et al., 2001) act as primary producers in this ecological context and can serve as Hg sources for the soil food web. Several studies have shown the relationship between THg concentration and the trophic level of terrestrial and aquatic animals (Crisol et al., 2008; Tsui et al., 2019; Yung et al., 2019; Monteiro et al., 2024). Similarly, the distribution of feeding habits across THg concentration groups revealed a general transition from a predominance of phytophagous and omnivorous organisms in the lower-concentration groups to a higher frequency of predators and organisms associated with decomposing organic matter in the higher-concentration groups (Fig. 4). This demonstrates that grouping organisms based on THg concentrations and feeding habits may be related to their ecological function. As organisms move up the trophic levels, THg concentration also increases (Figs. 4 and 5).

The grouping of organisms with the same feeding habit (Fig. 4) showed that group A (THg ≤ 43.50 ng g⁻^1^), represented by phytophagous species, and group B (43.50 < THg ≤ 112.92 ng g⁻^1^), represented by omnivores, had the lowest THg levels among the studied groups (range, 970.60 ng g⁻^1^). These invertebrates, predominant in group B, feed on both plant and animal matter (Brown et al., 2001), which increases their capacity to accumulate mercury. Cockroaches presented the highest frequency in group B (24 samples), which are opportunistic organisms feeding on both plant and animal resources (Grandcolas & Pellens, 2012).

Top predators in the soil food web showed a predatory feeding habit, which increases their mercury accumulation capacity (Hernández et al., 2022). This trend was confirmed in group C (THg > 192.62 ng g⁻^1^), which was predominantly composed of spiders (34 samples), terrestrial predatory animals. Beetles were the second most frequent group (31 samples), likely associated with the Staphylinidae family, which made up 30.43% of beetle samples and are mainly predators living under rocks, termite mounds, or ant nests (Fernandes et al., 2011; Lima, 1952). The Scarabaeidae family (20.65% of beetles) has detritivorous/necrophagous habits involving animal matter (Wink et al., 2005), supporting their high frequency in the group with the highest THg concentration (group C > 192.62 ng g⁻^1^; Fig. 4). The grouping of organisms by feeding habit in group C (112.92 < THg ≤ 273.81 ng g⁻^1^) and group D (THg > 273.81 ng g⁻^1^) also confirmed the trend previously observed by taxonomic grouping (Fig. 4).

The similarities and differences among feeding groups regarding their general THg accumulation patterns (Fig. 5) complement the classification based on organism frequencies (Fig. 4). Similar behavior regarding THg levels was observed among detritivores, necrophages, parasitoids, and predators (Fig. 5). This behavior may be related not only to the fact that apex predators consume more Hg (Tsui et al., 2019), but also to the bioavailability of mercury in decomposing tissues (necrophages/detritivores) or access to animal and plant excretions (parasitoids) (Rebolloso-Hernández et al., 2024).

Detritivores, necrophages, parasitoids, and predators exhibited partially similar profiles; this is likely associated with the fact that these organisms interact directly with decomposing organic matter, which can retain Hg and constitute a significant dietary exposure pathway, whereas predators acquire Hg through the consumption of contaminated prey (Monteiro et al., 2024; Tsui et al., 2019; Yung et al., 2019). In contrast, xylophagous and sap-sucking organisms were distinct from most other feeding groups yet showed similar patterns to one another, which may reflect their greater reliance on plant-derived resources. These results (Fig. 5) indicate that feeding habit contributes to Hg accumulation patterns but is not sufficient, by itself, to predict them since organisms occupying similar trophic positions may differ in THg concentration (Jelaska et al., 2014), BAF_Litter_, and body size.

## Strengths, limitations, and future directions

A major strength of this study is that it advances our understanding of Hg dynamics in epigeic soil fauna in the Brazilian Cerrado. To our knowledge, this is the first study to investigate the relationship between Hg concentrations in soil and litter and their accumulation in terrestrial soil invertebrates. Our findings also contribute to a better understanding of Hg cycling in the plant-soil interface of savanna ecosystems and provide a framework for future studies on the cycling of other trace metals with similar environmental behavior. A limitation of this study is the relatively coarse taxonomic resolution of the terrestrial invertebrates, as many specimens could not be identified to the genus or species level. This likely contributed to the high within-group variability in Hg concentrations, reflected by the large standard deviations, which in some cases exceeded the group mean. Another potential source of variation is gut content since whole-body specimens were homogenized for Hg analysis without prior depuration of the digestive tract. Despite this variability, the observed patterns of Hg bioaccumulation across trophic levels were consistent with biomagnification, reinforcing the value of terrestrial invertebrates as indicators of Hg transfer through terrestrial food webs. Future studies should prioritize species-level identification whenever possible and depurate the gastrointestinal tract before analysis to reduce variability associated with taxonomic heterogeneity and ingested material.

## Conclusions

Our results demonstrate that Hg dynamics in this terrestrial system are jointly controlled by environmental factors, such as organic matter and compartmental distribution, and biological traits, such as feeding habit and trophic position. We found significant differences in THg concentrations between soil and litter, with consistently higher levels in the litter compartment, confirming that this layer acts as a primary entry point and temporary reservoir of Hg in the system. Organic matter showed a significant positive influence on soil THg concentrations, whereas pH had no detectable effect, partially supporting our hypothesis and reinforcing the role of organic matter as a key regulator of Hg retention in Cerrado soils.

Surface-active soil invertebrates exhibited markedly higher THg concentrations than those observed in soil and litter, reaching values up to an order of magnitude greater, which demonstrates pronounced bioaccumulation. Bioaccumulation factors further confirmed that most taxa act as effective accumulators, with a predominance of macroaccumulation, particularly when soil is considered the exposure pathway. In addition, organisms could be consistently grouped according to THg concentrations and feeding habits, with trophic guilds sharing similar ecological traits exhibiting comparable accumulation patterns. Predatory organisms (top of the trophic level), such as spiders, exhibited higher THg concentrations compared to phytophagous organisms (base of the edaphic food chain) (e.g., Coleoptera: Chrysomelidae and Curculionidae) and generalist organisms (e.g., ants). Thus, the relationship between feeding habits and Hg concentrations not only enables the functional grouping of organisms but also reveals a trophic transfer process consistent with biomagnification. It is important to note that epigeic soil fauna have diverse and complex feeding behaviors, adaptable to the resources available in their microhabitat. Thus, although the literature often associates specific feeding habits with specific in surface-active soil invertebrates, such behavior may vary depending on environmental conditions. In this context, THg concentrations in these organisms may help delineate the trophic web structure established in different soil environments.

## Supplementary Information

Below is the link to the electronic supplementary material.

ESM 1 DOCX (406 KB)

## Data Availability

Data will be made available on request.
