# Supplementary material for: Mercury dynamics in surface-active soil invertebrates from an ecological reserve in the Brazilian savanna
Source: Environ Monit Assess. 2026 Oct 20;198(11):1230. doi: 10.1007/s10661-026-16051-0 (PMC13683486; doi:10.1007/s10661-026-16051-0)
Supplement: Supplementary file 1 [file 10661_2026_16051_MOESM1_ESM.docx]

**Supplementary Material 1**

The organisms were separated, counted, lyophilized, measured, and macerated using a mortar and agate pestle (Fig. S1). The samples from each point were composed of *n* individuals with the same taxonomic classification. The individuals were quantified and measured with a digital caliper, considering the distance from the tip of the head to the posterior end of the abdomen (legs and antennae were not considered). The average size (in mm) of the organisms was obtained by measuring at least three individuals per sample, except in cases where *n* < 3, in which all individuals were measured. After being macerated with an agate pestle, the samples were weighed and inserted into the equipment. This process was performed in triplicate for each sample. The diagram below details the processing of samples at each sampling point (Fig. S1).

**Fig. S1.** Sample processing diagram.

**Supplementary Material 2**

The classification tree was used to group the orders and feeding habits (descriptive classes) according to THg concentrations in the organisms (dependent variable). The classification was performed using the Chi-squared Automatic Interaction Detection (CHAID) algorithm, based on Pearson's chi-squared test. The classification tree results provide the number and frequency of observations in each group (range of THg concentrations), the proportion of observations corresponding to the predominant class of the dependent variable (purity), and the number of observations per descriptive class. Additionally, the confusion matrix presents the reclassification of observations according to the dependent variable, indicating which descriptive classes had the best classification among the groups.

The discriminant analysis was used to separate organisms by order, feeding habit, or sampling unit according to a set of three quantitative descriptors: THg concentrations in the organisms, average organism size (Hm), and the BAF calculated using THg concentrations in litter. The difference in the set of predictors among predefined descriptive classes (order, feeding habit, or sampling unit) was evaluated using Wilks' Lambda test. The Fisher distance matrix between the centroids of each group was calculated using Mahalanobis distance, allowing assessment of the significance of predictor differences between class pairs.

**Supplementary Material 3**

**References raised on the habit and functions of soil organisms described in Table 1**

Antunes, L., & Dionello, G. (2010). Bioecologia de *Sitophilus zeamais* Motschulsky 1885 (Coleoptera: Curculionidae).

Auad, A. M. (2003). Aspectos biológicos dos estágios imaturos de *Pseudodorus clavatus* (Fabricius) (Diptera: Syrphidae) alimentados com *Schizaphis graminum* (Rondani) (Hemiptera: Aphididae) em diferentes temperaturas. *Neotropical Entomology, 32*, 475–480.

Audino, L. D., Nogueira, J. M., da Silva, P. G., Neske, M. Z., Ramos, A. H. B., Moraes, L. D. M., & Borba, M. F. S. (2007). Identificação dos coleópteros (Insecta: Coleoptera) das regiões de Palmas (município de Bagé) e Santa Barbinha (município de Caçapava do Sul, RS).

Borror, D. J., & DeLong, D. M. (1988). *Introdução ao estudo dos insetos*. São Paulo: Edgard Blucher.

Brown, G. G., Fragoso, C., Barois, I., Rojas, P., Patrón, J. C., Bueno, J., Moreno, A. G., Lavelle, P., Ordaz, V., & Rodríguez, C. (2001). Diversidad y rol funcional de la macrofauna edáfica en los ecosistemas tropicales mexicanos. *Acta Zoológica Mexicana, número especial 1*, 79–110.

Camacho, G. P., & Feitosa, R. M. (2015). Estado da arte sobre a taxonomia e filogenia de Ectatomminae. In J. H. C. Delabie, R. M. Feitosa, J. E. Serrão, & C. S. F. Mariano (Orgs.), *As formigas poneromorfas do Brasil* (pp. 23–32). SciELO-Editus-Editora da UESC.

Correia, M. E. F., Aquino, A. M., & Aguiar-Menezes, E. D. L. (2008). Aspectos ecológicos dos isopoda terrestres.

Delabie, J. H. C., Feitosa, R. M., Serrão, J. E., Mariano, C. S. F., & Majer, J. D. (Eds.). (2015). *As formigas poneromorfas do Brasil*. SciELO-Editus-Editora da UESC.

DeLoach, C. J., Lewis, P. A., Herr, J. C., Carruthers, R. I., Tracy, J. L., & Johnson, J. (2003). Host specificity of the leaf beetle, *Diorhabda elongata deserticola* (Coleoptera: Chrysomelidae) from Asia, a biological control agent for saltcedars (*Tamarix*: Tamaricaceae) in the Western United States. *Biological Control, 27*(2), 117–147.

Estévez, A. L., & López Ruf, M. L. (2006). Separación de subfamilias en la familia de chinches Gelastocoridae (Hemiptera). *Revista de Biología Tropical, 54*(4), 1319–1322.

Fernandes, F. S., Alves, S. S., Santos, H. F., & Rodrigues, W. C. (2011). Staphylinidae e Silphidae (Coleoptera) como potenciais famílias bioindicadoras de qualidade ambiental. *Revista Eletrônica TECCEN, 4*(3), 17–32.

Hamada, N., Nessimian, J. L., & Querino, R. B. (2014). *Insetos aquáticos na Amazônia brasileira: taxonomia, biologia e ecologia*. Manaus: Editora do INPA.

Paarmann, W., Adis, J., Stork, N., Gutzmann, B., Stumpe, P., Staritz, B., ... & da Fonseca, C. R. (2001). The structure of ground beetle assemblages (Coleoptera: Carabidae) at fig fruit falls (Moraceae) in a terra firme rain forest near Manaus (Brazil). *Journal of Tropical Ecology, 17*(4), 549–561.

Rodrigues, S. M. M., Menezes, V. L., & Kaminski, E. (2011). Flutuação populacional do bicudo do algodoeiro em áreas de refúgio na intressafra. In *Anais do 8º Congresso Brasileiro de Algodão*. Campina Grande, PB: Embrapa Algodão.

Sottoriva, L. D. M., Picolo, L., Ramos, L. C. H., & Roel, A. R. (2007). Preferência alimentar e biologia reprodutiva de *Phibalosoma phyllinum* Gray, 1835 (Phasmatodea, Phasmatidae) em criações de laboratório. *Multitemas.*

Swift, M. J., Bignell, D., Moreira, F. M. S., & Huising, J. (2010). O inventário da biodiversidade biológica do solo: conceitos e orientações gerais. In F. M. S. Moreira, J. Huising, & D. E. Bignell (Orgs.), *Manual de biologia dos solos tropicais: amostragem e caracterização da biodiversidade* (pp. 23–41). Lavras: Editora da UFLA.

Todd, E. L. (1955). A taxonomic revision of the Family Gelastocoridae. *University of Kansas Science Bulletin, 37*, 277–475.

Weirauch, C., Bérenger, J. M., Berniker, L., Forero, D., Forthman, M., Frankenberg, S., ... & Zhang, J. (2014). An illustrated identification key to assassin bug subfamilies and tribes (Hemiptera: Reduviidae). *Canadian Journal of Arthropod Identification, 26*(2), 1–115.
